# Cognitive Profiles in Adolescents and Young Adults With Co‐Occurring Autism and First‐Episode Psychosis: A Preliminary Neuropsychological Investigation

**DOI:** 10.1002/pchj.70073

**Published:** 2026-01-26

**Authors:** Domily T. Y. Lau, Melody M. Y. Chan, Flora Y. M. Mo, Se‐Fong Hung, Kelly Y. C. Lai, Patrick W. L. Leung, Caroline K. S. Shea

**Affiliations:** ^1^ Alice Ho Miu Ling Nethersole Hospital, Hospital Authority Hong Kong Hong Kong Special Administrative Region China; ^2^ Tai Po Hospital, Hospital Authority Hong Kong Hong Kong Special Administrative Region China; ^3^ Queensland Brain Institute The University of Queensland St Lucia Queensland Australia; ^4^ Department of Psychiatry, Faculty of Medicine The Chinese University of Hong Kong Hong Kong Hong Kong Special Administrative Region China; ^5^ Department of Psychology The Chinese University of Hong Kong Hong Kong Hong Kong Special Administrative Region China

**Keywords:** autism, cognition, first‐episode psychosis, neuropsychology, psychiatric comorbidity

## Abstract

Autism spectrum disorder (ASD) and psychosis are traditionally considered distinct psychiatric conditions with divergent developmental trajectories, yet emerging evidence suggests they may share overlapping neurodevelopmental characteristics. This study examined whether the cognitive profile associated with co‐occurring autism and first‐episode psychosis (FEP) reflects additive or interactive influences of the two conditions. Neuropsychological profiles were compared across four age‐, sex‐, intelligence quotient‐, and education level‐matched groups of adolescents and young adults (*n* = 45; aged 13–21): individuals with co‐occurring ASD and FEP (FEP‐ASD), FEP without ASD (FEP‐O), ASD without FEP, and non‐autistic controls. The FEP‐ASD group exhibited an uneven cognitive profile characterised by relative strengths in visuospatial processing and recognition memory, alongside marked impairments in information processing speed, attentional control, and working memory. This pattern resembled the ASD profile but at a lower overall performance level, consistent with the additive impact of psychosis on ASD‐related cognitive characteristics. FEP‐ASD participants outperformed FEP‐O in recognition memory, a domain usually preserved in ASD but impaired in psychosis. These preliminary findings suggest that co‐occurring ASD and psychosis may produce a cognitive profile shaped by influences from both conditions. Larger longitudinal and multimodal studies are needed to clarify the underlying mechanisms.

## 1 Introduction

Our understanding of psychiatric comorbidity is still in its infancy (Nordgaard et al. 2023). One particularly underexplored intersection lies between autism spectrum disorder (ASD) and psychosis. Converging epidemiological studies suggest that ASD‐psychosis comorbidity is far more common than previously assumed (Hallerbäck et al. 2012; Larson et al. 2017; Mandell et al. 2012; Waris et al. 2013). In Hong Kong, for example, nearly 30% of adolescents with first‐episode psychosis (FEP) also have a diagnosis of ASD (Kwok et al. 2024). Importantly, longitudinal data also suggest a developmental link, with ASD diagnosis significantly predicting later psychotic experiences in youth (Sullivan et al. 2013). Taken together, these findings indicate a meaningful relationship between autism and psychosis.

Understanding this comorbidity is not merely of academic interest but has important clinical implications. A longitudinal study reported that adolescents and young adults with FEP who present with elevated autistic features have higher psychosis symptom severity across 2 years of follow‐up (Pelizza et al. 2025). Another longitudinal study further demonstrated that high autistic traits at baseline predicted poorer clinical trajectories over 1 years (Zheng et al. 2021). Other cross‐sectional studies also showed that high autistic traits in patients with psychosis are strongly associated with poorer social functioning (Ziermans et al. 2021), increased depression, hopelessness, and suicidal risk (Upthegrove et al. 2018). These findings underscore the importance of recognising co‐occurring autistic characteristics and psychosis, as individuals with both conditions may have greater symptom severity and more complex support needs.

Despite these clinical implications, the nature of ASD‐psychosis comorbidity remains unresolved. Two major conceptual models have been proposed to account for this comorbidity: one suggests that ASD‐psychosis represents an autism‐based presentation with additional psychotic features—essentially reflecting the additive impact of psychosis on an underlying autistic phenotype (Larson et al. 2017), while the other posits that autistic traits may compensate for some functional impairments typically associated with psychosis, suggesting an interactive rather than purely additive profile (Abu‐Akel et al. 2022). These frameworks have important implications for care because ASD and psychosis typically follow different clinical pathways (Larson et al. 2017). ASD is typically supported through behavioural and psychosocial interventions, whereas psychosis is primarily treated with antipsychotic medications. Understanding how ASD intersects with psychosis is therefore essential for guiding appropriate assessment and tailoring interventions to individual needs.

Given these competing conceptual models, it is reasonable to empirically show how ASD and psychosis jointly shape cognitive functioning. Neuropsychology offers a valuable and relatively low‐cost approach to advance this understanding by characterising the cognitive profiles associated with ASD‐psychosis comorbidity. As suggested by previous meta‐analytic evidence (Kuo and Eack 2020), while adults with ASD and FEP show overlapping cognitive deficits, they also exhibit key differences: individuals with ASD tend to outperform those with psychosis in visuospatial tasks, visual attention, working memory, and nominative language, while both groups show comparable impairments in information processing and language comprehension. These distinctions suggest that cognitive profiling may help disentangle ASD‐psychosis comorbidity. If ASD and psychosis are associated with partially dissociable cognitive profiles, two possibilities arise. Under an additive account, co‐occurring ASD and psychosis should produce a combined profile in which ASD‐related strengths are preserved, while psychosis‐related impairments are superimposed. Under a compensatory/interactive account, ASD‐related cognitive characteristics may buffer some psychosis‐related impairments, leading to cognitive patterns that deviate from the simple sum of the two profiles.

However, it remains unclear whether co‐occurring ASD and psychosis yield a cognitive profile that reflects an additive combination of both conditions, or an interactive modification of one by the other. Existing studies (e.g., Bechi et al. 2020; Deste et al. 2018; Deste et al. 2020; Vaskinn and Abu‐Akel 2019) focus on the contribution of autistic traits to the cognitive heterogeneity observed in psychosis but do not directly assess individuals with both ASD and psychosis as a distinct clinical group. To address this important but ignored gap, the current study aimed to (1) examine whether adolescents with comorbid ASD and FEP show a combined cognitive profile reflecting influence from both diagnoses, and (2) characterise which aspects of the cognitive profile resemble ASD, psychosis, or are shared across both conditions.

To achieve these aims, we conducted a cross‐sectional study comparing cognitive performance across four groups: adolescents with (1) comorbid FEP and ASD (FEP‐ASD), (2) FEP without ASD (FEP‐O), (3) ASD without psychosis (ASD), and (4) typically developing (TD) controls. To minimise the confounding effects of disease chronicity on cognitive performance, we limited our psychosis sample to individuals who were recently discharged from acute hospitalisation for their FEP and were in a clinically stabilised phase. When they were hospitalised, diagnoses were established by a multidisciplinary child and adolescent psychiatry team, based on structured interviews, clinical observations, standardised ASD/psychosis screening tools, and parent‐report information. Participants completed a comprehensive neuropsychological battery covering both basic and higher‐order cognitive domains: visuospatial processing, recognition memory, immediate and delayed recall, processing speed, attentional control, working memory, and mentalising.

## 2 Methods

### 2.1 Participants

This study was approved by the Joint Chinese University of Hong Kong—New Territories East Cluster Clinical Research Ethics Committee (Reference number: CRE‐2022.238). Table 1 summarises the four diagnostic groups and the abbreviations used throughout this work. We hypothesised that the FEP‐ASD group would exhibit a combined cognitive profile reflecting influences from both ASD and psychosis. To evaluate this, we tested for a group × cognitive domain interaction across the four groups (FEP‐ASD, FEP‐O, ASD, and TD). Based on a previous meta‐analysis by Kuo and Eack (2020), the observed effect sizes representing the differences between autism and psychosis in non‐social cognition were in the medium range (Hedge's *g* = 0.33–0.64). Given that the cognitive domains assessed by our neuropsychological battery are functionally distinct but may be influenced by shared mechanisms (Barch and Sheffield 2014), we assumed a correlation coefficient of 0.5 for the repeated measures. Using G*Power 3.1.9.6 software (Faul et al. 2009), we estimated that a total sample of 44 participants (*n* = 11 per group) would be required to detect a statistically significant (*α* = 0.05) within‐between interaction with 80% power, based on a medium effect size of *f* = 0.2.

**TABLE 1:** Overview of participant groups and diagnostic composition.

Participant group	Abbreviation	Autism diagnosis	Psychosis diagnosis
Patients diagnosed with first‐episode psychosis and autism spectrum disorder	FEP‐ASD	Yes	Yes
Patients diagnosed with first‐episode psychosis only	FEP‐O	No	Yes
Patients diagnosed with autism spectrum disorder only	ASD	Yes	No
Typically developing individuals	TD	No	No

**FIGURE 1:**
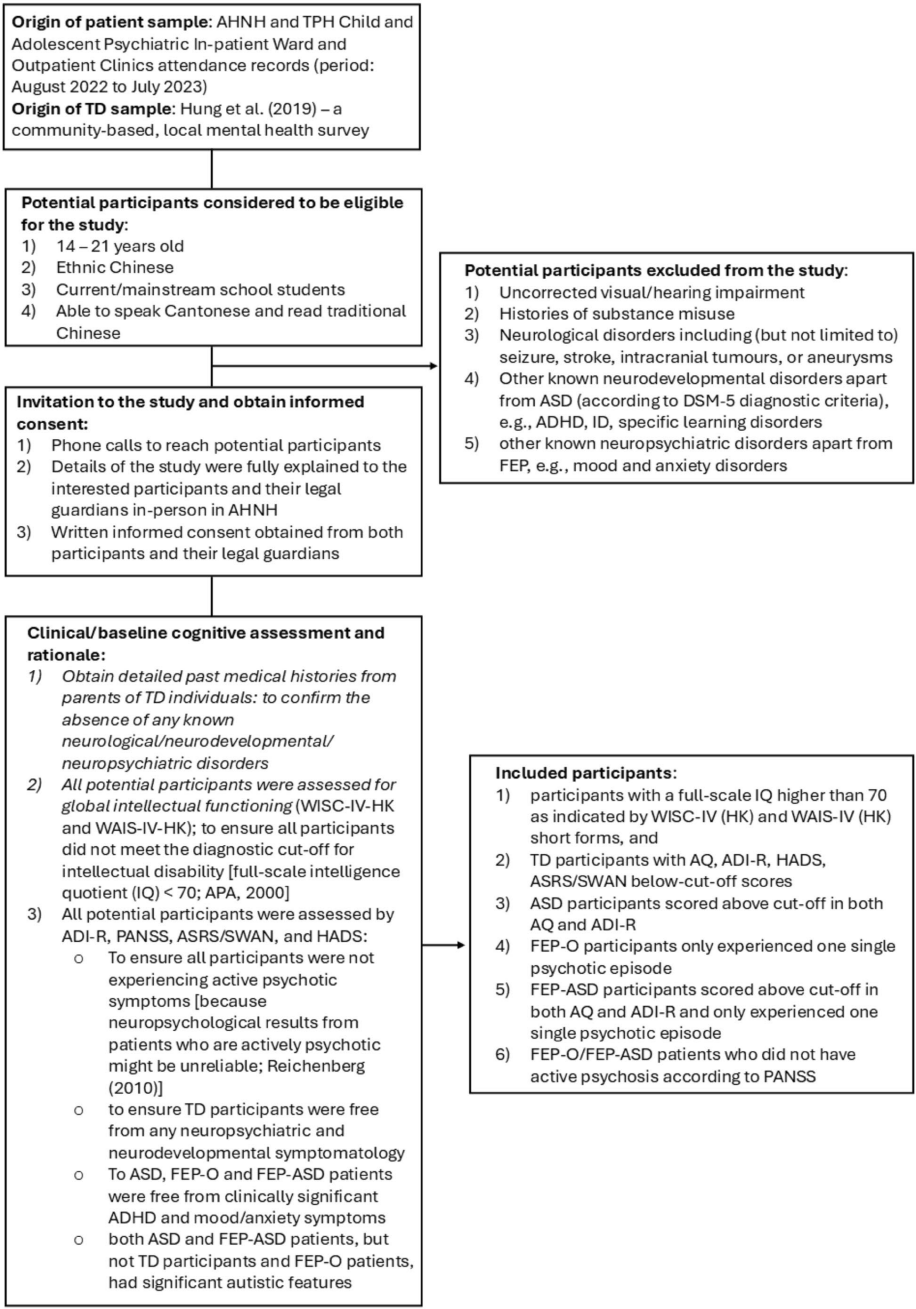
A flowchart illustrating participant inclusion and exclusion procedure. People with FEP were defined as individuals who had a functional psychotic disorder fulfilling DSM‐5 criteria with first treatment contact (Breitborde et al. 2009), including Schizophrenia, Schizoaffective disorder, Schizophreniform disorder, Bipolar I disorder (manic or depressed episode) with psychotic features, Major Depressive Disorder with psychotic features, Delusional Disorder, Other Specified Schizophrenia Spectrum and Other Psychotic Disorder, Unspecified Schizophrenia Spectrum and Other Psychotic Disorder.

Detailed inclusion and exclusion criteria are outlined in Figure 1. Participants with ASD, FEP‐O, and FEP‐ASD were identified from inpatient and outpatient service records between August 2022 and July 2023. Demographic and diagnostic information was extracted from the Clinical Management System (CMS) and Clinical Data Analysis and Reporting System (CDARS). All diagnoses were made by a consultant‐led multidisciplinary team (K.L., F.M., S.F.H., and C.S.) according to DSM‐5 criteria [see Kwok et al. 2024 for details]. TD participants were selected from a community‐based prevalence survey and screened using the Diagnostic Interview Schedule for Children—Version 5 (DISC‐5). Those with a history of neurodevelopmental or psychiatric conditions were excluded.

### 2.2 Procedure and Measures

Following informed consent, the first author conducted a clinical and cognitive screening session with each participant and their caregiver. Participants' general cognitive ability was estimated using the Hong Kong version of the Wechsler Intelligence Scale for Children—Fourth Edition short form (WISC‐IV[HK]) for participants under 16 years, and the Wechsler Adult Intelligence Scale—Fourth Edition short form (WAIS‐IV[HK]) for those aged 16 and above. Current psychotic symptoms were screened using the Positive and Negative Syndrome Scale (PANSS); individuals scoring above 58 were excluded (Leucht et al. 2005).

Neurodevelopmental and psychiatric profiles were assessed using the Autism Diagnostic Interview—Revised [ADI‐R; Lord et al. 1994], the Adult ADHD Self‐Report Scale [ASRS; Kessler et al. 2005], the Strengths and Weaknesses of ADHD Symptoms and Normal Behaviour Scale—Parent Version [SWAN; Chan et al. 2014], and the Hospital Anxiety and Depression Scale [HADS; (Chan et al. 2010)]. Medical histories of TD participants were obtained from parents. Psychosis symptomatology for participants with FEP (with/without ASD) was extracted from CMS, CDARS, and patient files. This included psychosis subtype (affective vs. non‐affective), age of onset, duration of untreated psychosis, illness duration, and details of psychotropic medications.

All participants completed a fixed‐sequence, paper‐and‐pencil neuropsychological battery (Table 2) assessing eight cognitive domains. With the exception of processing speed and mentalising, each domain included both verbal and nonverbal tasks. Assessments were administered individually in a quiet clinical room by the first author, who was trained and supervised by a clinical psychologist. Sessions lasted approximately 2.5 h.

**TABLE 2:** A list of neuropsychological tests used in this study.

Cognitive domain	Verbal			Nonverbal		
	Test name (Reference)	Brief description	Parameter	Test name (Reference)	Brief description	Parameter
*Visuospatial processing*	/			Block design [WISC‐IV(HK); Wechsler 2003 and WAIS‐IV(HK); Wechsler 2008]	Timed task recreating a two‐dimensional pattern using red/white blocks in the three‐dimensional space	No. of correct response (Standard Score)
*Recognition memory*	The Hong Kong List Learning Test (Chan and Kwok 1999)	Hear a list of 16 Chinese words through three trials; immediately (immediate recall) and after 30 min (delayed recall), participants are asked to recall as many words as possible; recognition trial: participants hear individual words and are asked to indicate whether each word has appeared in the original word list	No. of correct response (raw scores for each condition)	Rey‐Osterrieth Complex Figure Test [RCFT; Meyers and Meyers 1995]	Copy and reconstruction of figure after 3 min (immediate recall) and 30 min (delayed recall); recognition trial after delayed recall task	No. of correct response (raw scores for each condition)
*Immediate recall*						
*Delayed recall*						
*Processing speed*	/			(Children's) Color Trails Test (CCTT/CCT) form A (D'Elia et al. 1996; Williams et al. 1995)	Chaining a sequence of numbers (CCTT/CCT‐1)	Time needed for task completion (Standard Score)
				Symbol‐digit modalities test [WISC‐IV(HK); Wechsler (2003) and WAIS‐IV(HK); Wechsler (2008)]	Timed task, pair specific numbers with given geometric figures	Time needed for task completion (Standard Score)
*Attentional control*	Victoria Stroop Color‐Word Test, Hong Kong Version (Lee et al. 2002)	See a colour word printed with the same/different colour; say the colour of the word but not what the word says	No. of errors (color‐word condition)	d2 Test of Attention [d2; Brickenkamp and Zilmer 1998]	Timed task marking target symbol (letter d with two dashes) among 658 items in total	Concentration performance (correctly marked items‐ errors)
				(Children's) Color Trails Test (CCTT/CCT) form B (D'Elia et al. 1996; Williams et al. 1995)	Chaining a sequence of alternating numbers and colors (CCTT‐2/CCT‐2) that are randomly distributed on a single paper	Time needed for task completion (Standard Score)
*Working memory*	Backward digit Span [WISC‐IV(HK); Wechsler (2003) and WAIS‐IV(HK); Wechsler (2008)]	Hear a sequence of digits then immediately verbally repeat the sequence in reverse order	No. of spans completed (Standard Score)	/		
*Mentalising*	/			Reading the Mind in the Eyes test (RMET; Baron‐Cohen et al. 2001)	Visually presented with sets of eyes and some words; point to the word that best describes what the person in the picture is thinking or feeling	% correct

### 2.3 Data Analysis

Statistical analyses were performed using SPSS version 29.0. Demographic and clinical characteristics of all participants were compared using Chi‐square tests (or Fisher's Exact Test, FET, when assumptions were violated) for categorical variables. For continuous variables, one‐way analysis of variance (ANOVA) was used for multiple‐group comparisons. Comparisons of psychosis symptomatology between the FEP subgroups (FEP‐ASD and FEP‐O) were conducted using independent samples *t*‐tests. For the neuropsychological data, participants' scores on all tests were first converted to *z*‐scores based on the means and standard deviations of the non‐autistic group. Where multiple tests were used to assess a cognitive domain, the *z*‐scores were averaged to yield a single composite score for that domain. To compare neuropsychological performance across the four groups, a 4 × 8 mixed factorial ANOVA was conducted, with group identity as the between‐subject factor and cognitive domain as the within‐subject factor. If Mauchly's test of sphericity was significant, Greenhouse–Geisser corrections were applied and reported. When significant interaction and main effects were observed, Bonferroni‐adjusted *post hoc* comparisons were performed.

## 3 Results

### 3.1 Demographic and Clinical Characteristics

**FIGURE 2:**
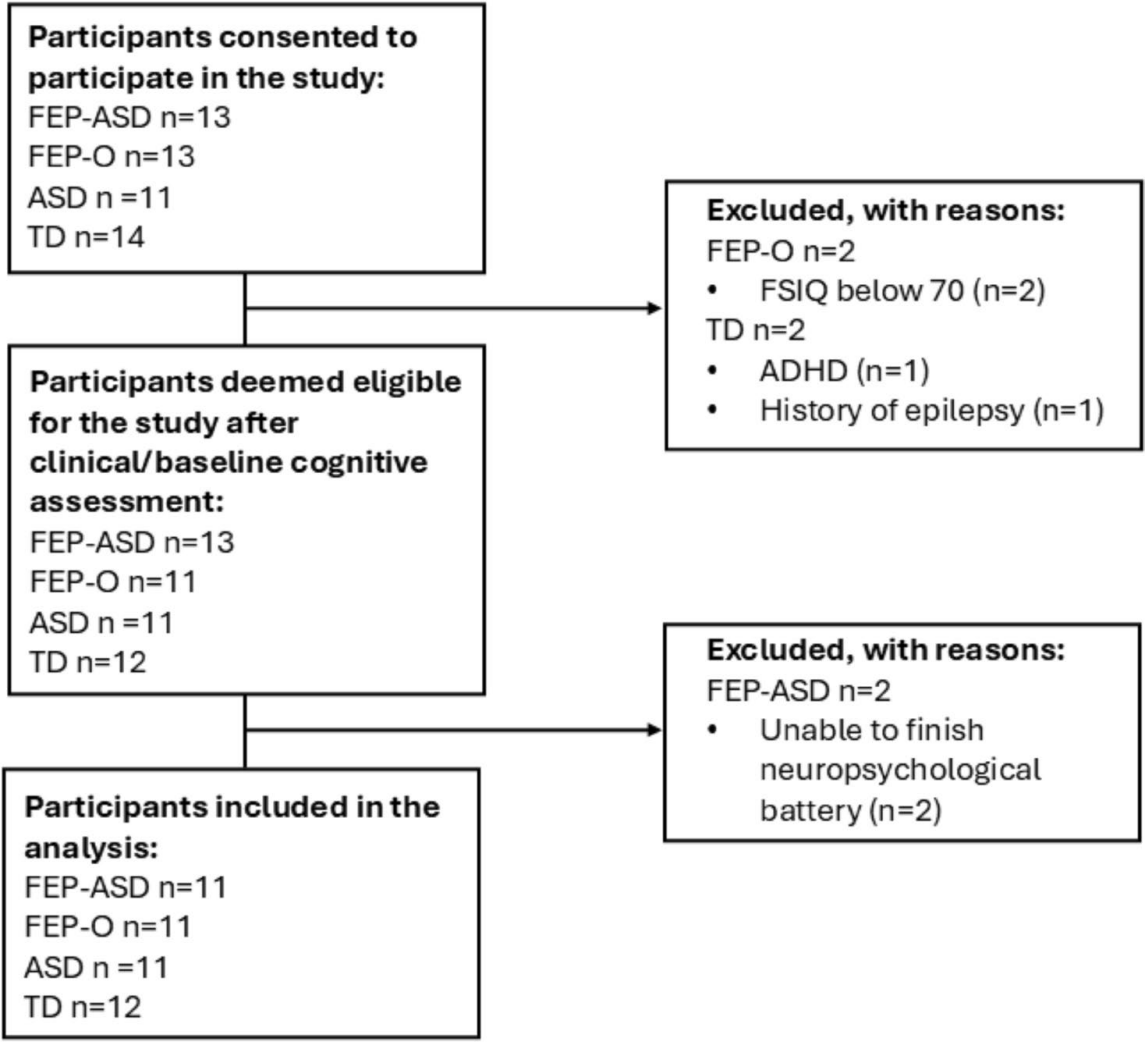
A flowchart illustrating the number of participants included in different stages of the recruitment process.

From August 2022 to July 2023, 51 individuals and their caregivers provided written informed consent to participate in the study. Following screening, four participants were excluded, and two were unable to complete the neuropsychological assessment. As a result, data from 45 participants were included in the final analysis. A flowchart outlining the participant recruitment process is presented in Figure 2.

Table 3 summarises the demographic and clinical characteristics of the four participant groups. The FEP‐ASD, FEP‐O, ASD, and TD groups were comparable in terms of sex, age (15.6–16.7 years), intelligence quotient (full‐scale IQ = 91–103), and years of education (9.6–10.9 years). Regarding psychosis symptomatology (Table 4), the FEP‐ASD and FEP‐O groups showed broadly similar clinical characteristics.

**TABLE 3:** Participants' demographic and clinical characteristics.

Subgroup	FEP‐ASD (*n* = 11)	FEP‐O (*n* = 11)	ASD (*n* = 11)	TD (*n* = 12)	Statistics (F *χ* ^2^)	Omnibus test *p* value; post hoc pairwise comparisons
Demographic details						
Sex, *n*					*χ* ^2^ = 0.073	< 1.00
Male	5	5	5	6		
Female	6	6	6	6		
Age (Years), mean (s.d.)	15.55 (2.50)	16.73 (2.45)	15.64 (2.42)	16.08 (1.62)	F_3,41_ = 0.63	0.60
Education (Years), mean (s.d.)	9.64 (2.25)	10.91 (2.21)	9.91 (2.47)	10.50 (1.17)	F_3,41_ = 0.85	0.47
Clinical characteristics						
Full‐scale IQ mean (s.d.)	92.09 (6.91)	90.91 (11.52)	99.73 (9.17)	103.25 (18.52)	F_3,41_ = 2.59	0.066
PANSS total score, mean (s.d.)	46.82 (8.72)	40.73 (7.32)	36.45 (3.14)	32.42 (2.15)	F_3,41_ = 12.24	< 0.001***; *FEP‐ASD>ASD* ** *;* *FEP‐ASD>TD* *** *;* *FEP‐O>TD* *
ADI‐R‐A (Reciprocal social interactions) (s.d.)	20.82 (5.15)	5.45 (3.90)	18.36 (6.28)	3.25 (2.86)	F_3,41_ = 40.68	< 0.001***; *FEP‐ASD>FEP‐O* *** *;* *FEP‐ASD>TD* *** *;* *FEP‐O<ASD* *** *ASD>TD* ***
ADI‐R‐B (Communications) (s.d.)	12.91 (2.84)	4.27 (2.53)	14.82 (4.14)	3.50 (2.88)	F_3,41_ = 40.68	< 0.001***; *FEP‐ASD>FEP‐O* *** *;* *FEP‐ASD>TD* *** *;* *FEP‐O<ASD* *** *ASD>TD* ***
ADI‐R‐C (RRB) (s.d.)	4.64 (1.75)	0.55 (0.93)	6.91 (2.91)	1.00 (0.95)	F_3,41_ = 31.53	< 0.001*** *FEP‐ASD>FEP‐O* *** *;* *FEP‐ASD>ASD* * *;* *FEP‐ASD>TD* *** *;* *FEP‐O<ASD* *** *ASD>TD* ***
HADS (Depressive mood) (s.d.)	8.00 (3.41)	7.00 (4.83)	3.45 (2.25)	5.25 (3.02)	F_3,41_ = 3.62	0.021* *FEP‐ASD>ASD* *
HADS (Anxiety symptoms) (s.d.)	10.27 (5.42)	6.18 (3.82)	5.64 (3.56)	6.58 (2.31)	F_3,41_ = 3.21	0.033* *FEP‐ASD>ASD* *

*Note:* All pairwise comparisons are Bonferroni‐corrected.

* *p* < 0.05.

** *p* < 0.01.

*** *p* < 0.001.

**TABLE 4:** Psychosis symptomatology of participants with FEP.

	FEP‐ASD (*n* = 11)	FEP‐O (*n* = 11)	Statistics (χ^2^/LHR/t)	*p*
Affective, *n*			χ^2^ = 1.63	0.20
Affective	4	7		
Non‐affective	7	4		
Age of onset (Years), mean (s.d.)	13.73 (2.01)	15.27 (2.01)	*t* = −1.81	0.086
DUP (Months), mean (s.d.)	10.00 (8.15)	9.81 (11.73)	*t* = 0.042	0.97
Duration of illness (Months), mean (s.d.)	22.55 (14.96)	20.00 (14.06)	*t* = 0.411	0.69
Antipsychotics use, *n*	12	12 (100)		
Atypical, *n*	12	12 (100)		
Typical, *n*	0	0 (0)		
Number of antipsychotics use, mean (s.d.)	1.27 (0.47)	1.18 (0.41)	*t* = 0.49	0.63
Use of olanzapine/clozapine			FET = 1.27	0.58
Olanzapine or clozapine used, *n*	3	1		
Other atypical antipsychotics	8	10		
Antidepressant use (Y:N), *n*	5:6	5:6	*χ* ^2^ < 0.001	> 0.99
Mood stabilizer use (Y:N), *n*	5:6	1:10	FET = 3.92	0.15
Benzodiazepines use (Y:N), *n*	1:10	2:9	FET = 0.39	> 0.99
Benzhexol use (Y:N), *n*	2:9	4:7	FET = 0.93	0.64

Abbreviation: FET = fisher's exact test.

### 3.2 Neuropsychological Test Performance

**FIGURE 3:**
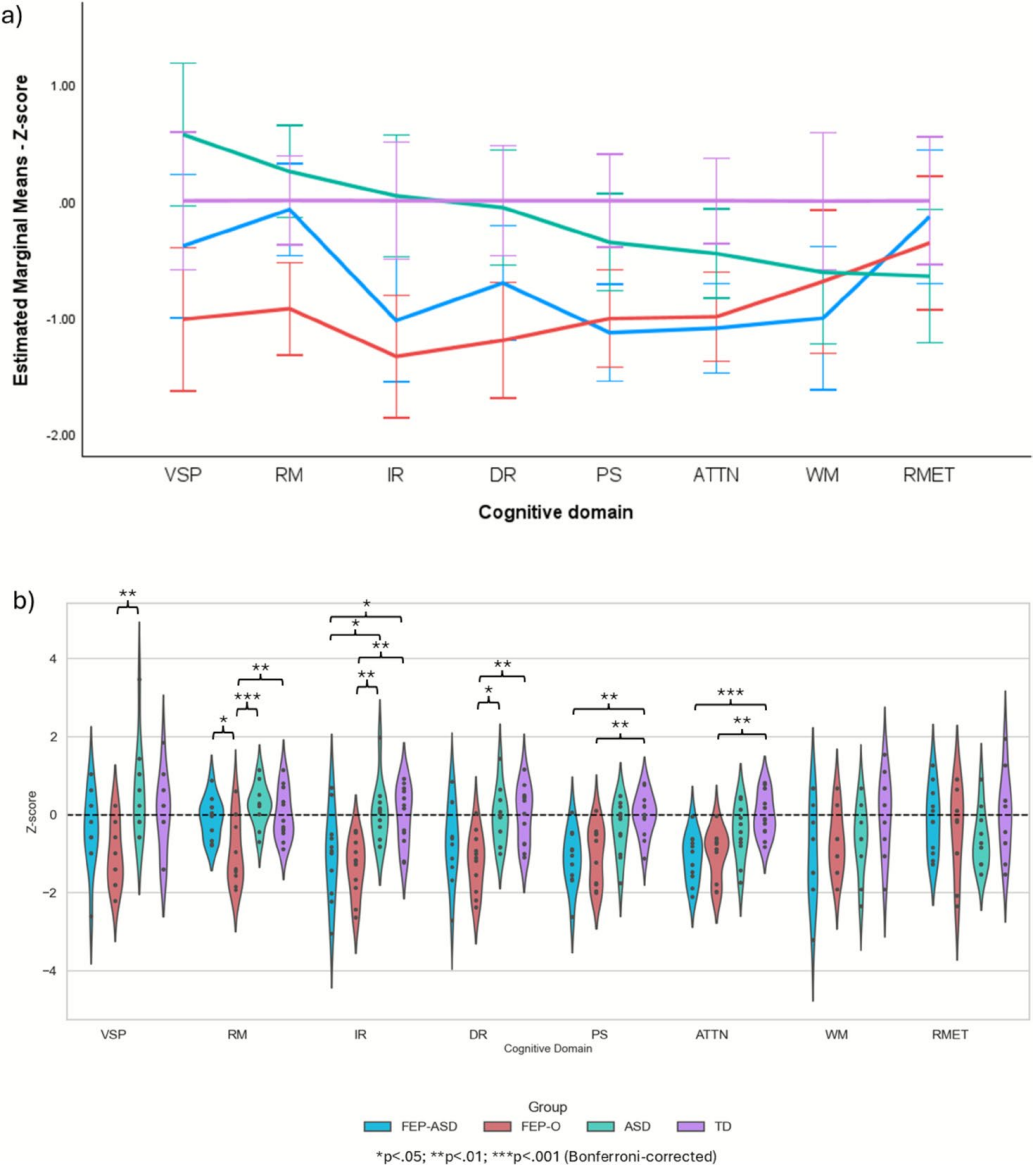
Neuropsychological test performance. Panel a illustrates the significant interaction effect. Panel b is a violin plot showing individual variability as well as the post hoc pairwise comparison results. ATTN = attentional control; DR = delayed recall; IR = immediate recall; PS = processing speed; RM = recognition memory; RMET = Reading the Mind in the Eyes test tapping mentalising; VSP = visuospatial processing; WM = working memory.

Participants' neuropsychological test performance is illustrated in Figure 3, and the descriptive statistics (mean, standard error, 95% confidence interval) are reported in Table S1. A mixed factorial ANOVA revealed a significant group × cognitive domain interaction effect (*F*_14.66,200.41_ = 2.10, *p* = 0.012, η_p_^2^ = 0.133, Figure 3a), as well as a significant main effect of group (*F*_3,41_ = 11.23, *p* < 0.001, η_p_^2^ = 0.451). Post hoc Bonferroni‐corrected pairwise comparisons reveal a pattern of domain‐specific group differences (Figure 3b). The FEP‐ASD group exhibited an uneven cognitive profile, with relative strengths in recognition memory and visuospatial processing, but significant impairments in processing speed (*p* < 0.01) and attentional control (p < 0.001) compared to the TD group. Despite overall lower performance, FEP‐ASD participants outperformed people with FEP‐O in recognition memory (*p* < 0.05). In contrast, the FEP‐O group showed widespread cognitive impairment, with significantly lower scores than TD across six of eight domains (all *p* < 0.05). The ASD group displayed a statistically nonsignificant but characteristically uneven profile, with preserved performance in perceptual and memory domains and relative weakness in executive processes. Together, these results suggest that the cognitive profile observed in FEP‐ASD may reflect the additive impact of ASD‐related strengths and psychosis‐related impairments, rather than a compensatory interaction between the two conditions.

## 4 Discussion

It remains unknown whether individuals with co‐occurring ASD and psychosis show characteristic cognitive performance patterns that distinguish them from individuals with ASD or psychosis alone. This preliminary study yielded two key observations: (1) adolescents with FEP‐ASD demonstrated a characteristic cognitive profile consistent with the additive influence of ASD‐related strengths and psychosis‐related difficulties; and (2) FEP‐ASD participants performed significantly better than the FEP‐O group in recognition memory.

FEP‐ASD individuals displayed an uneven cognitive profile. Specifically, their performance on tasks assessing perceptual reasoning, recognition memory, and delayed recall was comparable to that of their ASD and TD counterparts—domains in which FEP‐O participants were significantly impaired. In contrast, FEP‐ASD participants showed marked deficits in tasks requiring executive control (i.e., fluency, attentional control, and working memory), with performance levels significantly lower than those of ASD and TD participants, but comparable to FEP‐O. This uneven profile closely resembles the cognitive characteristics typically reported in ASD populations (Black et al. 2009; Melling and Swinson 2016). By comparison, FEP‐O individuals in our sample exhibited global impairments across both basic and higher‐order domains, consistent with existing literature (Catalan et al. 2021; Seidman et al. 2016). The similarity in cognitive profiles between FEP‐ASD and ASD suggests that ASD‐related strengths may be preserved in some domains despite the presence of psychosis, whereas impairments in higher‐order cognition are more consistent with the cognitive disruptions typically observed in psychosis. This interpretation aligns with an additive account of co‐occurring ASD and psychosis (Larson et al. 2017), although an equally plausible interpretation is that the cognitive characteristics observed in FEP‐ASD may simply reflect transient cognitive disruption associated with acute psychosis superimposed on an ASD cognitive profile, rather than evidence for a stable neurodevelopmental profile. Determining whether any unique mechanisms exist will require longitudinal studies incorporating genetic, neurophysiological, or neuroimaging data.

Not only can individuals with FEP‐ASD be distinguished from those with FEP‐O based on their overall cognitive profile, but they can also be differentiated by performance in specific cognitive domains. In particular, FEP‐ASD participants performed significantly better than FEP‐O participants in recognition memory—a domain in which their performance was comparable to that of individuals with ASD and TD controls. This finding aligns with previous studies showing that individuals with psychosis typically exhibit impaired recognition memory relative to TD individuals (Pelletier et al. 2005), while individuals with ASD tend to demonstrate intact recognition memory (Desaunay et al. 2020).

These findings have potential clinical relevance. Individuals with comorbid ASD and psychosis are at higher risks of poorer quality of life, social functioning (Chisholm et al. 2019), and slower symptom improvement (Zheng et al. 2021), yet diagnosis is complicated by overlapping symptomatology and reliance on subjective assessments prone to bias (Sampson et al. 2021; Trevisan et al. 2020). Neuropsychological profiling may offer a useful complementary tool to improve diagnostic accuracy (Schalbroeck et al. 2023), though formal testing is often impractical in routine settings. This proof‐of‐concept study highlights domain‐specific patterns in FEP‐ASD that may be useful for guiding future research on assessment approaches in this population.

4.1

Although this study—with carefully selected and well‐matched psychotic, autistic, and non‐autistic groups—offers valuable insights into this specific comorbid population, the findings remain exploratory and are not yet sufficient to inform clinical decision making.

The primary limitation of this study is its modest sample size, the potential for sampling bias, and the constraints of a cross‐sectional design. Although some observed effect sizes were large (Table S1), these are likely inflated due to sampling variability and potential model overfitting. Approximately half of the cognitive domains demonstrated medium‐to‐large effects, which may serve as a preliminary reference for power calculations in future research on FEP‐ASD and related neurodevelopmental conditions; however, these results should be interpreted with caution. Moreover, participants with FEP in the present study were clinically stabilised, had relatively short DUP, and were recruited shortly after hospital discharge. This may represent a subset of adolescents with milder psychosis. As such, the cognitive patterns observed may not generalise to individuals with a more severe clinical profile. Also, the cross‐sectional study design implies that all interpretations regarding additive versus interactive effects are necessarily speculative; the present findings cannot determine whether the observed pattern reflects stable cognitive characteristics or transient changes associated with an acute psychotic episode. Given the substantial inter‐ and intra‐individual variability observed within the FEP‐ASD group (Figure 3b) and the possibility of sampling bias, larger multicentre replication studies—ideally with longitudinal follow‐up—will be essential to establish the stability and generalisability of the observed cognitive patterns.

Another important limitation relates to the potential impact of clinical variables on cognition. Although the FEP groups (i.e., FEP‐ASD and FEP‐O) were matched on key clinical variables (e.g., medication type, DUP, residual symptoms), we cannot preclude the possibility that individual differences in these factors may still influence cognitive performance. While accumulating evidence in FEP indicates minimal differences in cognitive impact between commonly prescribed atypical antipsychotic medications such as risperidone and olanzapine (Keefe et al. 2007; Cuesta et al. 2009), DUP (Ito et al. 2015) and residual symptoms (Leeson et al. 2009) are nevertheless known to modulate cognitive functioning and ideally should be controlled for statistically. Given the small sample, including these as covariates was not feasible without risking overfitting, but future studies should account for these potential confounders explicitly.

Even so, the present study provides important initial evidence that may inform future genetic, cognitive neuroscience, and clinical research. Continued investigation is needed to advance our understanding of how best to identify and support individuals with co‐occurring FEP and ASD—an under‐recognised subgroup associated with poorer prognosis and a need for sustained multidisciplinary care.

## Funding

The authors have nothing to report.

## Conflicts of Interest

The authors declare no conflicts of interest.

## Supporting information


**Table S1:** Neuropsychological test performance—descriptive statistics.

## Data Availability

The data that support the findings of this study are available on request from the corresponding author. The data are not publicly available due to privacy or ethical restrictions.
