# Supplementary material for: Cognitive Profiles in Adolescents and Young Adults With Co‐Occurring Autism and First‐Episode Psychosis: A Preliminary Neuropsychological Investigation
Source: Psych J. 2026 Jan 26;15(1):e70073. doi: 10.1002/pchj.70073 (PMC12834706; doi:10.1002/pchj.70073)
Supplement: Supplementary file 1 — Table S1: Neuropsychological test performance—descriptive statistics. [file PCHJ-15-e70073-s001.docx]

**Table S1: Neuropsychological test performance – descriptive statistics**

| **Group** | **Domain** | **Mean** | **Standard Error** | **95% Confidence Interval** | |
| --- | --- | --- | --- | --- | --- |
|  |  |  |  | Lower Bound | Upper Bound |
| **FEP-ASD** | *VSP* | -.388 | .305 | -1.003 | .227 |
|  | *RM* | -.075 | .197 | -.473 | .322 |
|  | *IR* | -1.027 | .260 | -1.552 | -.502 |
|  | *DR* | -.705 | .245 | -1.201 | -.210 |
|  | *PS* | -1.131 | .206 | -1.547 | -.714 |
|  | *ATTN* | -1.093 | .190 | -1.476 | -.710 |
|  | *WM* | -1.007 | .304 | -1.622 | -.393 |
|  | *RMET* | -.136 | .283 | -.708 | .436 |
| **FEP-O** | *VSP* | -1.017 | .305 | -1.633 | -.402 |
|  | *RM* | -.925 | .197 | -1.323 | -.528 |
|  | *IR* | -1.335 | .260 | -1.860 | -.810 |
|  | *DR* | -1.197 | .245 | -1.693 | -.702 |
|  | *PS* | -1.011 | .206 | -1.427 | -.594 |
|  | *ATTN* | -.995 | .190 | -1.377 | -.612 |
|  | *WM* | -.693 | .304 | -1.307 | -.078 |
|  | *RMET* | -.362 | .283 | -.934 | .210 |
| **ASD** | *VSP* | .569 | .305 | -.046 | 1.184 |
|  | *RM* | .251 | .197 | -.146 | .648 |
|  | *IR* | .042 | .260 | -.483 | .567 |
|  | *DR* | -.058 | .245 | -.554 | .437 |
|  | *PS* | -.356 | .206 | -.773 | .060 |
|  | *ATTN* | -.453 | .190 | -.836 | -.070 |
|  | *WM* | -.613 | .304 | -1.227 | .002 |
|  | *RMET* | -.647 | .283 | -1.219 | -.075 |
| **TD** | *VSP* | 3.886e-16 | .292 | -.589 | .589 |
|  | *RM* | .002 | .188 | -.378 | .383 |
|  | *IR* | .001 | .249 | -.502 | .503 |
|  | *DR* | .001 | .235 | -.474 | .475 |
|  | *PS* | .001 | .197 | -.398 | .400 |
|  | *ATTN* | .001 | .181 | -.366 | .367 |
|  | *WM* | -.002 | .291 | -.591 | .586 |
|  | *RMET* | 1.110e-16 | .271 | -.548 | .548 |

Note:

VSP=visuospatial processing; RM=recognition memory; IR=immediate recall; DR=delayed recall; PS=processing speed; ATTN=attentional control; WM=working memory; RMET=mentalising
